# Financial Risk Prediction and Entrepreneurs’ Psychological Status Under Entrepreneurial Psychology

**DOI:** 10.3389/fpsyg.2021.750917

**Published:** 2022-01-11

**Authors:** Xiao Liang, Ying Yang, Wenxi Ruan, Ji Liu, Bo Zhang, Zheng Xu, Shaojun Xu

**Affiliations:** ^1^Shanxi VC/PE Fund Management Co., Ltd, Taiyuan, China; ^2^Northeast Asian Studies College, Jilin University, Changchun, China; ^3^Taizhou Vocational College of Science and Technology, Taizhou, China; ^4^School of Electrical and Information Engineering, University of Sydney, Sydney, NSW, Australia; ^5^China Asset Management Co., Ltd., Beijing, China; ^6^Chinese Academy of Fiscal Sciences, Beijing, China; ^7^Center for Quantitative Economics, School of Business, Jilin University, Changchun, China

**Keywords:** financial risk prediction, new entrepreneurs, entrepreneurial psychological states, psychological capital, questionnaire

## Abstract

Entrepreneurship plays an important role in the development of national economy. The study aims to accelerate the construction of social and economic structure by improving the success rate of new entrepreneurs in the process of innovation and entrepreneurship. First, the related theories of financial risk prediction are introduced, and entrepreneurial psychological status and the psychological states on entrepreneurship are analyzed. Second, the current situation of entrepreneurial psychology of new entrepreneurs is analyzed through a questionnaire survey and model test. The results show that private enterprises account for the largest proportion, with a percentage of 58.14% of 125. In total, 32 Chinese-foreign joint ventures rank second and account for 14.88% of the total, and the scale of each type of enterprises accounts for 25%, respectively. The operating years of enterprises are mainly between 10 and 20 years. Among the enterprises surveyed, the significant level of entrepreneurial psychology and entrepreneurial opportunity of entrepreneurs is *p* = 0.000–0.01. It indicates that males’ psychological adjustment ability and entrepreneurial ability in the entrepreneurial process are higher than females’, and their entrepreneurial psychological states and entrepreneurial ability will improve with the growth of age, education levels, and positions. It is concluded that entrepreneurial psychological capital and entrepreneurial opportunity are significantly positively correlated with financial risk expectation. The research results prove the impact of financial risks on the entrepreneurial psychology of new entrepreneurs, and provide a reference for new entrepreneurs in predicting financial risks.

## Introduction

Generally, the construction of an economic structure based on innovation and entrepreneurship requires the support of the government and social institutions. However, compared with the entrepreneurs in developed countries, Chinese entrepreneurs have to play two roles, that is, they have to create economic miracles and adapt themselves to the Chinese market economy system (Belás et al., 2018; Mashrur et al., 2020), which brings them great pressure. How to get Chinese entrepreneurs out of the pressure and promote the development of the entrepreneurial economy is a problem faced by ministries, entrepreneurs, and scholars (Hermansson and Cecilia, 2018; Yao and Qin, 2021). At present, the research on how to establish an entrepreneurial economy mainly focuses on macroeconomic policies, ignoring the culture and policies of a region (D’Amico et al., 2018; Ding, 2020). Therefore, it is necessary to rationally allocate resources and build a real entrepreneurial economy of new enterprises. Otherwise, the entrepreneurial economy will not play a part in spreading the political thoughts (Sukor et al., 2021; Zeb et al., 2021). Then, how to encourage the new entrepreneurs rationally allocate resources and improve their performance are two problems to be solved by decision-makers and entrepreneurs.

If positive psychological and organizational behavior are wanted under the entrepreneurial economy, the following questions should be answered: What are positive psychological behavior and positive organizational behavior under the entrepreneurial economy? Is psychological capital more important than human capital and social capital and does it become an advantage in the competition? Does entrepreneurs’ psychological capital affect the profitability of new enterprises? How do entrepreneurs develop their psychological capital and improve their performance in developing the entrepreneurial economy? The above questions will be answered here. Studying entrepreneurship from the perspective of entrepreneurs is a typical research paradigm (Zhang et al., 2020; Guo, 2021).

New entrepreneurs are taken as the research subject, and their entrepreneurial psychological states based on financial risk prediction are summarized after the analysis of the results of a questionnaire survey and a model test. The innovation is to combine financial risk prediction and entrepreneurial psychology of new entrepreneurs. The study provides a reference for the research on the influence of the psychological states of new entrepreneurs on their entrepreneurial success.

## Literature Review

The concept of psychological capital first appears in the literature of economic experts. Garcia et al. (2018) argued that psychological capital is viewed as the personalized characteristics of a person, including self-view, self-esteem, attitudes toward work, ethical orientation and outlooks on life (Garcia et al., 2018). Hadi and Abdullah (2018) used the term “psychological capital” in their study of gender inequality and pointed out that the economic returns of organizational commitments and expectations reflected the psychological capital (Hadi and Abdullah, 2018). Psychologist Baskaran (2018) pointed out that one of the main shortcomings of psychological research in the 20th century is that it lacks attention to people’s positive qualities. Before that, mainstream psychology mainly emphasizes people’s psychological problems and negative emotions, and psychology becomes pathological or negative psychology (Baskaran, 2018). Pailot et al. (2018) advocated the research on psychology should focus on the positive aspects rather than the negative, which brings a positive change to the study on psychology. Positive psychology opens a new perspective to interpret people’s psychological states. It focuses on psychological health and positive psychological states. It is a science aimed at promoting the development and self-realization of individuals, groups and the whole society (Pailot et al., 2018). Also, the professors and colleagues of the American Gallup Organization defined psychological capital and started to study it as a central concept. Hu et al. (2018) discussed the theoretical origin, construction dimension and practical significance of human resource management, which lays a solid foundation for subsequent empirical research (Hu et al., 2018). In short, the concept of psychological capital is determined according to the basis and standard of positive organizational behavior. Positive psychological states, such as self-efficacy, hope, optimism and resilience, are the research objects of positive organizational behavior. Psychological capital broadens the thoughts of researchers and triggers a lot of discussions.

The dimensions of entrepreneurial psychological capital have strong correlation, but their concentrations are different. Entrepreneurial self-efficacy puts emphasis on the positive self-cognitive characteristics of entrepreneurs. Entrepreneurial hope and optimism attach importance to the positive attribution model in the face of challenges and failures. Entrepreneurial resilience highlights the cognitive reconstruction and emotional recovery after entrepreneurial activities. And the sub-dimensions of entrepreneurial psychological capital plays a synergistic role, that is, its overall effect is greater than the sum of all parts (Chavan and Meena, 2018; Shu et al., 2018). Also, psychological capital can reflect individuals’ positive psychological strength under the theoretical framework of positive psychological and organizational behavior. It is a kind of psychological resources and the result of long-term interaction between individuals and the environments, and can be strengthened through training. Different from traditional human capital and social capital, it rests on knowledge, skills, relationships and connections, making individuals release their pressure from the external environment by using these psychological resources, which can evoke human potential and develop their competitive advantages (Ahsan et al., 2018; Nindyasari et al., 2021).

## Theories of Psychological States and Entrepreneurial Psychological States

### Financial Risks

The internal causes of financial risks are: (1) Due to the imbalance of the structure of the financial system, the bank system with indirect financing of some financial institutions and their asset-liability periods do not match. (2) The blind encouragement of financial innovation leads to loose the supervision and management to the relevant financial institutions and products, especially to the cross-industry and cross-border financial products from various asset management activities and important financial holding companies, resulting in risk transfer between different financial sectors and institutions. (3) Some financial activities and bank credit deliberately escape from financial regulation and violate the requirements for capital adequacy ratio and deposit-loan ratio, which weakens the roles of macroeconomic regulation and control and worsens the financial system. (4) The financial institutions don’t want to pay off the risk cost, which adds the burden to the central bank. (5) Induced by investors, financial institutions are easy to be involved in high-risk activities.

Financial risks usually exist in the areas where the economy is under transformation (Petit et al., 2018). First, excess capacity, high debt ratio, the great gap between monetary growth and economic growth, and other potential risks affect the sustainable and healthy development of the financial industry. Second, the public risk brings the risk in the financial system in the transitional period, and the increasing debts of the local government hinder the development of banks and other financial institutions (Qian et al., 2018; Chen, 2019). Third, some policies of commercial banks on making profits, increasing liquidity, and preventing risks change with the change of the interest rate, and the reform of the foreign exchange system and capital accounts, and the internationalization of RMB lead to more domestic and international risks.

### Source of Entrepreneurial Psychological States

The structure and connotation of psychological capital are complementary. Enriching the connotation of psychological capital, strengthening its assistance ability and updating its structure are helpful to understanding the connotation of psychological capital, and they are also the elements to develop psychological capital. According to its components, psychological capital can be divided into economic capital, human capital, and social capital (Rogoza et al., 2018; Wu and Song, 2019). Economic capital is tangible asset, including people’s funds and wealth. Human capital, also known as intellectual capital, involving the knowledge, skills, and cognitive ability acquired by individuals through education or experience (Wu et al., 2019; Wu Y. J. et al., 2020). Social capital refers to the communication between individuals, labor relations network, and mutual trust. At present, there are a lot of studies on the positive impact of economic capital, human capital, and social capital on job performance and entrepreneurial success. The results show that with the rapid development of the world economy and the intensified competition among enterprises, more and more managers and scholars realize that they do not have enough money to develop economic, human and social capital, and they are still trying to improve corporate performance and maintain their competitive advantages in a traditional way. In this case, many managers gradually realize that employees’ positive psychological resources play an important role in improving the organizational competitive ability in practice. Afterward, the impact of employees’ positive psychological resources on job performance and positive organizational behavior is heatedly discussed in academia by empirical experiments (Wu W. et al., 2020; Yuan and Wu, 2020). Zhou and Wu (2018) proposed that the research on positive psychology should focus on the important role of human positive psychology (Zhou and Wu, 2018). Here, psychological capital is studied, hoping that it can become the competitive advantage of individuals and organizations. The research provides a reference for the research on improving the competitive ability of enterprises.

### Entrepreneurial Psychological Model Based on Entrepreneurial Opportunity

Some experts believe that there are three ways to study entrepreneurial opportunities, namely, survival concept, structural concept, and structural concept. The concept emphasizes the fact that entrepreneurial opportunities already exist in the market, especially profit opportunities. The idea is that the relationship structure between individuals or organizations in a specific state generated by entrepreneurial opportunities, especially a supervisory and information advantage, is relative to other individuals or organizations. The constructed idea is that entrepreneurial opportunities do not exist independently, but are created or constructed by people in the interaction with the environment. Some experts divide entrepreneurial opportunities into identified, discovered, and created opportunities based on their clarity of means and objectives. Identifying opportunities means that entrepreneurs can identify opportunities through the relationship between means and objectives when the relationship between means and objectives in the market is very obvious. The discovery of opportunities means that the state of any party is more than the opportunity for entrepreneurs to explore unknown and expected possibilities. Creativity is not targeted, and opportunities should be more insightful and entrepreneurial than others in creating market opportunities (Wu and Wu, 2017; Zheng et al., 2018). The most popular definition of entrepreneurial opportunities is that new products, new services, new raw materials, and new organizational methods can be introduced and sold at higher production costs. Some experts believe that entrepreneurial opportunities are feasible and profitable enterprises that provide innovative products or services to the market or imitate profitable products or services in an unsaturated market. In short, the possibility of entrepreneurship is a possibility to meet the needs. In other words, entrepreneurial opportunities must be tested in the market and have the potential of endurance. Industry opportunity has its market position, value chain, and competitive prospect.

Psychological capital is based on the existing theory and research of human capital, capitalism, and social capital, and is beyond economic capital, human capital, and social capital. It has a fundamental position and value of entrepreneurs, especially when strategic decisions need to be made timely, and high risk and uncertainty are dealt with. The psychological capital of entrepreneurs is more important. Entrepreneurs and service agencies seeking to support entrepreneurship believe that psychological capital is an important individual difference, although psychological capital and economic capital, human capital, and social capital are key factors for enterprise success. Shen et al. (2019) believed that psychological capital was the most competitive forward-looking capital, which can coordinate human, economic, and social capital effectively, achieving success in entrepreneurship (Shen et al., 2019).

**FIGURE 1:**
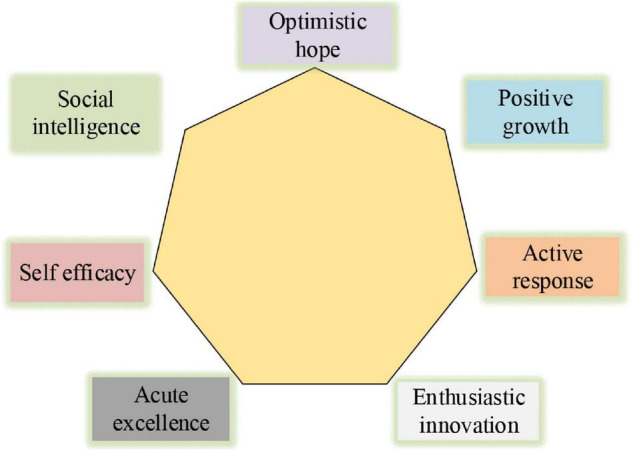
Entrepreneurial psychological model of new entrepreneurs.

Entrepreneurial psychological capital is the extension of the concept of psychological capital. It is the psychological resources and quality of groups under specific circumstances, especially the psychological capital of entrepreneurial entrepreneurs. Some experts find that enterprise psychological capital is a model with seven factors, including optimistic hope, sensitivity and excellence, positive growth, positive adaptation, enthusiasm and innovation, self-efficacy, and social ability. The specific model is shown in Figure 1 below. Active adaptation mainly refers to how to overcome difficulties and setbacks. Enthusiasm innovation mainly refers to a life full of passion and vitality, living a difficult life, daring to do it, and feeling their vitality. Efficiency mainly refers to the multi-task processing ability, namely the social wisdom to know others and their motivation and emotion to do appropriate things in different situations, as well as the strong communication ability. Optimistic characters mainly refer to an optimistic and calm mentality, as well as the will to achieve the goal.

## Research Methods of Entrepreneurial Psychological States of New Ventures Based on Financial Risk Prediction

The research on the entrepreneurial psychological states of new entrepreneurs based on financial risk prediction is carried out by an online questionnaire survey. The questionnaire survey is conducted on the “questionnaire star” platform. A total of 250 questionnaires are distributed and 215 valid questionnaires are recovered. For the recovered questionnaires, SPSS 25.0 is used for reliability and validity analysis, and the results show that the designed questionnaire has good reliability and validity. The data of the questionnaire survey show the overall situation of the entrepreneurial psychology of new entrepreneurs under financial risk prediction, and excel mathematical statistics software is used to analyze the collected data. The questionnaire has two parts. One is the basic information of enterprises and the other is the current situation of entrepreneurial psychological states of new entrepreneurs. In the questionnaire, entrepreneurial psychological states include optimistic characters, social wisdom, self-efficacy, acute excellence, passionate innovation, active response, and positive growth, and the key questions in the questionnaire are shown in Table 1.

**TABLE 1:** Key questions in the questionnaire.

Number	Questions
Q1	Your gender?
Q2	Your age?
Q3	Your education level?
Q4	Your position?
Q5	Enterprise size?
Q6	Enterprise nature?
Q7	Operating years?
Q8	Enterprise industry?
Q9	I can analyze the problem and find a solution.
Q10	I can overcome the challenges.
Q11	I am a man who dares to do what is asked to do.
Q12	In any case, I have an optimistic attitude.
Q13	If I find myself in trouble, I can think of many ways to get out of it.
Q14	You can stick to your goals even when you are depressed
Q15	I can take very important business opportunities.
Q16	In the entrepreneurial process, I can establish and maintain relationships through various channels.
Q17	I like to enjoy the process of achieving goals.
Q18	I can enrich my knowledge regularly.
Q19	I can quickly know about customers’ needs.
Q20	At work, I can handle a lot of things at the same time.

The internal consistency reliability coefficient of the entrepreneurial psychology is tested, as shown in Table 2 below.

**TABLE 2:** Consistency coefficient of entrepreneurial psychology.

Indicators	Optimistic hope	Social intelligence	Self-efficacy	Acute excellence	Enthusiastic innovation	Active response	Positive growth	Total amount
Cronbach a coefficient	0.799	0.767	0.843	0.804	0.868	0.878	0.816	0.967

The consistency reliability of internal indicators in Table 2 above is 0.967, and the reliability of indicators is greater than 0.8, indicating that the reliability of the scale is good.

## Analysis on Entrepreneurial Psychological States of New Ventures Based on Financial Risk Prediction

### Basic Information of New Entrepreneurs

**FIGURE 2:**
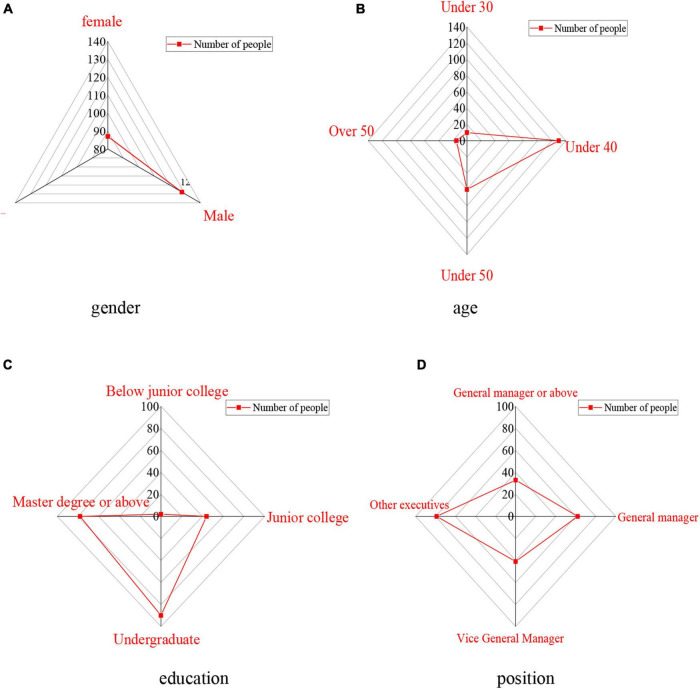
Basic information of entrepreneurs. **(A)** Gender statistics results. **(B)** Age statistics results. **(C)** Education statistics results. **(D)** Position statistics results.

**FIGURE 3:**
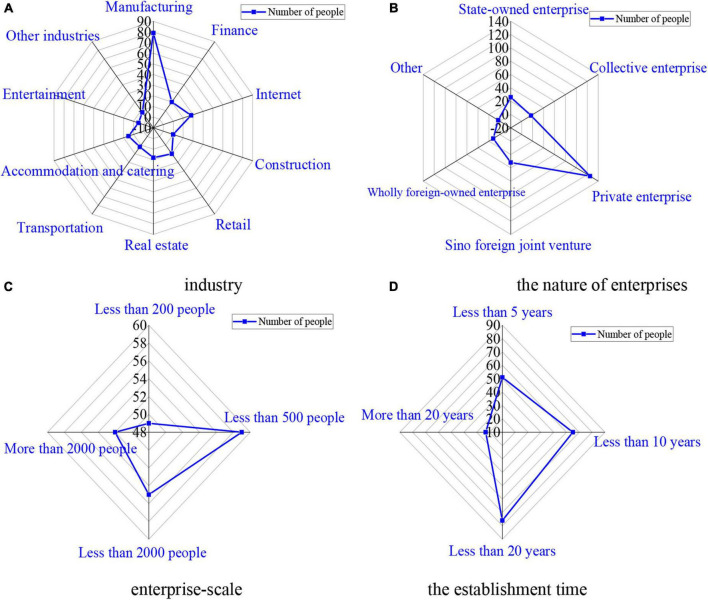
Basic information of enterprises. **(A)** Industry statistics results. **(B)** The nature of enterprises statistics results. **(C)** Enterprise-scale statistics results. **(D)** The establishment time statistics results.

The basic information of the subjects is introduced, including gender, age, education level, position, industry, the nature of enterprises, the scale of enterprises, and the operation years of enterprises, as shown in Figures 2, 3.

Figure 2 shows that the research subjects include 87 females and 128 males. The ages of them are between 30 and 50 years old. Among the entrepreneurs surveyed, 90 have bachelor’s degrees followed by 78 with master’s degrees. In terms of their positions, the subjects who are general managers account for 28.8% and other executives take up more than 36.7%.

Figure 3 shows that the industries in which the subjects are employed are extensive, including the financial industry, the Internet industry, the construction industry, the retail industry, the real estate industry, the transportation industry, the accommodation and catering industry, the entertainment industry, and other industries. Among them, the study on the impact of psychological states on systemic financial risks shows that the samples of this survey are representative. As for the nature of enterprises, private enterprises account for the largest proportion of 58.14%, with a total of 125, followed by 32 Sino-foreign joint ventures of 14.88%. The data and the proportion are compared, and the results show that the data are valid and reliable. For the scale of enterprises, the survey sample groups includes a group with less than 200 people, a group with less than500 people, a group with less than 2000 people, and a group with more than 2000 people, and the proportion of the scale of each type of enterprises is close to 25%. For the operation years of enterprises, 78 enterprises have been run for more than 10 years but less than 20 years, accounting for 36.28%, followed by 65 enterprises run for less than 10 years. Overall, the survey samples are representative.

### Entrepreneurial Psychologies of New Entrepreneurs Based on Financial Risk Prediction

**FIGURE 4:**
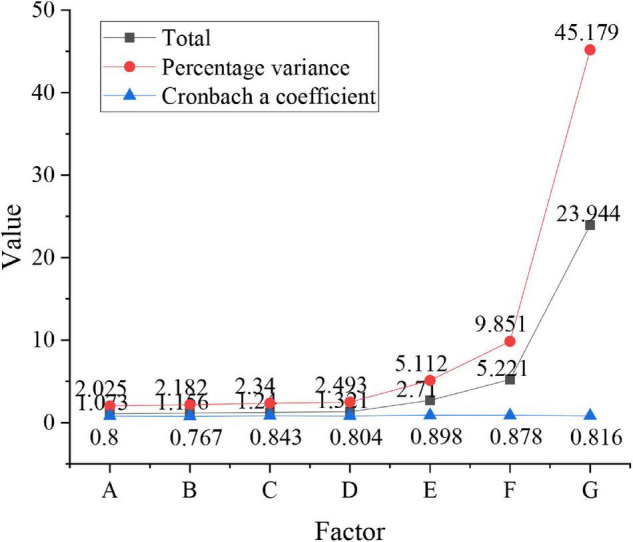
Consistency coefficient and exploratory factor analysis of the entrepreneurial psychologies of new entrepreneurs.

The entrepreneurial psychologies of new entrepreneurs are studied from (A), social ability (B), self-efficacy (C), acute excellence (D), passion for innovation (E), active coping (F), positive growth (G), and their exploratory factor analysis is shown in Figure 4 below.

**FIGURE 5:**
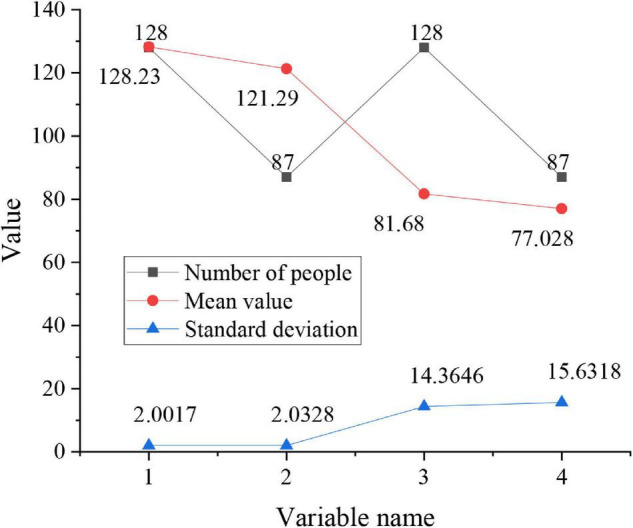
*T*-test between gender and entrepreneurial psychological states.

Figure 4 shows that the consistency coefficient involved is 0.967, and the reliability of social ability is 0.767, and the consistency reliability of other factors is greater than 0.8. The eigenvalues of seven entrepreneurial psychologies are greater than 1, explaining 69.186% of all variables. This indicates that the reliability of the psychological scale of entrepreneurship proposed is good and can be used. The relationship between gender and entrepreneurial psychological states is shown in Figure 5.

**FIGURE 6:**
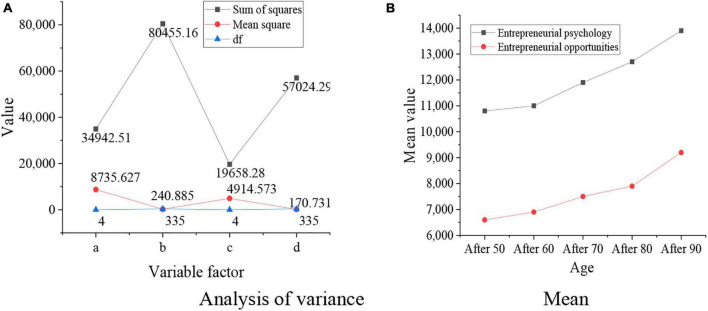
Relationship between the ages of entrepreneurs and their entrepreneurial psychological and entrepreneurial ability. **(A)** Analysis of variance results. **(B)** Mean results.

In Figure 5, 1 represents the entrepreneurial psychological states of males, 2 represents the entrepreneurial psychological states of females, 3 represents the entrepreneurial abilities of males, and 4 represents the entrepreneurial abilities of females. Among them, the entrepreneurial psychological states *t* = 3.467 (*p* < 0.01) and the entrepreneurial ability *t* = 2.834 (*P* < 0.01), which shows that the entrepreneurial psychological states of males are better than those of the females, and the entrepreneurial opportunities of males are more than those of females. Therefore, it can be said that the entrepreneurial psychological states and entrepreneurial capital of entrepreneur are affected by genders. The relationship between the ages of entrepreneurs and their entrepreneurial psychological states and entrepreneurial ability is shown in Figure 6.

**FIGURE 7:**
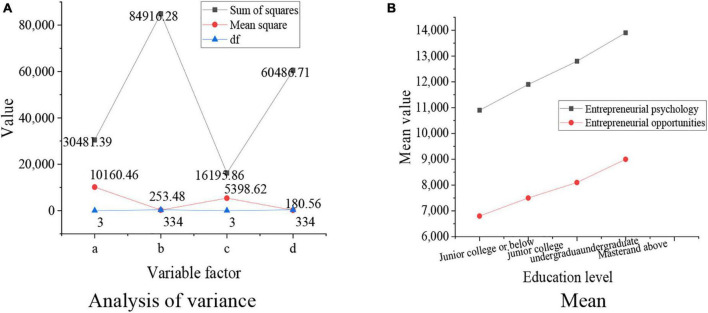
Relationship between the educational levels of entrepreneurs and their entrepreneurial opportunities and abilities. **(A)** Analysis of variance results. **(B)** Mean results.

In Figure 6A shows the entrepreneurial psychological states of the groups, b shows the entrepreneurial psychological states within the groups, c shows the entrepreneurial ability of the groups, and d shows the entrepreneurial ability of the groups. The result is that *p* = 0.000 < 0.01, which indicates that entrepreneurial psychological states and entrepreneurial capital of entrepreneurs are affected by their ages. Entrepreneurial psychological states and entrepreneurial ability will be improved with the growth of ages. The relationship between the educational levels of entrepreneurs and their entrepreneurial opportunities and abilities is shown in Figure 7 below.

**FIGURE 8:**
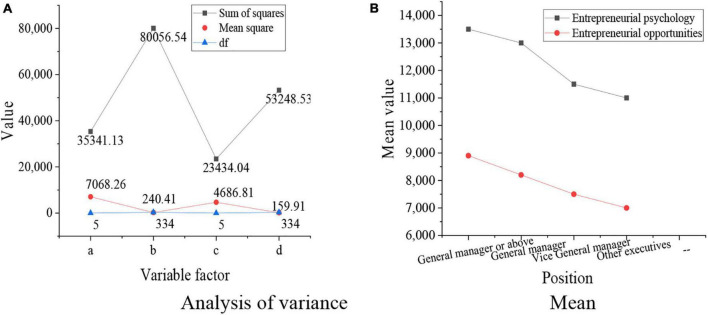
Relationship between the positions of entrepreneurs and the entrepreneurial opportunity and ability. **(A)** Analysis of variance results. **(B)** Mean results.

Figure 7 shows that *p* = 0.000, which is less than 0.01, indicating that the entrepreneurial psychological states and entrepreneurial capital of entrepreneurs are affected by their educational levels. Entrepreneurial psychological states and entrepreneurial ability of entrepreneurs will be improved if they have higher educational levels. The relationship between the positions of entrepreneurs and their entrepreneurial opportunities and entrepreneurial abilities is shown in Figure 8 below.

Figure 8 shows that *p* = 0.000, which is less than 0.01, indicating that the entrepreneurial psychological states and entrepreneurial ability of entrepreneurs are affected by their positions. The lower the positions of the entrepreneurs are, the worse their entrepreneurial psychological states and entrepreneurial abilities are.

In short, private enterprises account for the largest proportion, accounting for 58.14%, with a total of 125, followed by 32 Chinese-foreign joint ventures of 14.88%. The scale of each type of enterprises accounts for 25%, respectively. The operation years of these enterprises are concentrated between 10 and 20 years, accounting for 36.28%. In the enterprises investigated, their entrepreneurial psychology and entrepreneurial opportunity ability are significant, that is, *p* = 0.000 < 0.01, indicating that males’ psychological adjustment ability and entrepreneurial ability are higher than females’ in the entrepreneurial process. The entrepreneurial psychological states and entrepreneurial ability will be improved with the increase of age, educational levels and positions. It is concluded that entrepreneurial psychological capital and entrepreneurial opportunity ability are significantly positively correlated with financial risk prediction.

## Conclusion

Based on financial risks, the basic theories of new entrepreneurs and psychology are studied. Further research is conducted by analyzing the characteristics of new entrepreneurs. The model is verified by a questionnaire survey, and the influencing factors and current situation of entrepreneurial psychology of new entrepreneurs are analyzed. The results show that private enterprises account for 73.02% of the total number of Chinese-foreign joint ventures, and entrepreneurial psychology and entrepreneurial ability are dynamically affected by various factors. The entrepreneurial psychology and entrepreneurial opportunity ability of entrepreneurs is *p* = 0.000 < 0.01, and males’ psychological adjustments and entrepreneurial ability is higher than females’. With the continuous increase of educational levels, ages and positions, the mean of entrepreneurial psychology and entrepreneurial ability is increasing. It is concluded that entrepreneurial psychological capital is positively correlated with entrepreneurial opportunity ability. The innovation is to analyze the psychological change process of new entrepreneurs under the financial risk, which provides a reference for related research. Therefore, the research of this paper has an important impact on the entrepreneurial success of new entrepreneurs to analyze entrepreneurial psychology and entrepreneurial ability. However, there are still some limitations. The size of samples is small and is not sufficient to represent all new entrepreneurs. Therefore, in the follow-up study, the sample size will be expanded.

## Data Availability Statement

The raw data supporting the conclusions of this article will be made available by the authors, without undue reservation.

## Ethics Statement

The studies involving human participants were reviewed and approved by Jilin University Ethics Committee. The patients/participants provided their written informed consent to participate in this study. Written informed consent was obtained from the individual(s) for the publication of any potentially identifiable images or data included in this article.

## Author Contributions

All authors listed have made a substantial, direct, and intellectual contribution to the work, and approved it for publication.

## Conflict of Interest

XL was employed by Shanxi VC/PE Fund Management Co., Ltd. BZ was employed by China Asset Management Co., Ltd. The remaining authors declare that the research was conducted in the absence of any commercial or financial relationships that could be construed as a potential conflict of interest.

## Publisher’s Note

All claims expressed in this article are solely those of the authors and do not necessarily represent those of their affiliated organizations, or those of the publisher, the editors and the reviewers. Any product that may be evaluated in this article, or claim that may be made by its manufacturer, is not guaranteed or endorsed by the publisher.
